# Supplementary material for: Classification of COVID-19 Patients into Clinically Relevant Subsets by a Novel Machine Learning Pipeline Using Transcriptomic Features
Source: Int J Mol Sci. 2023 Mar 3;24(5):4905. doi: 10.3390/ijms24054905 (PMC10002748; doi:10.3390/ijms24054905)
Supplement: Supplementary file 1 [file ijms-24-04905-s001.zip › Supplemental Figures.pdf]

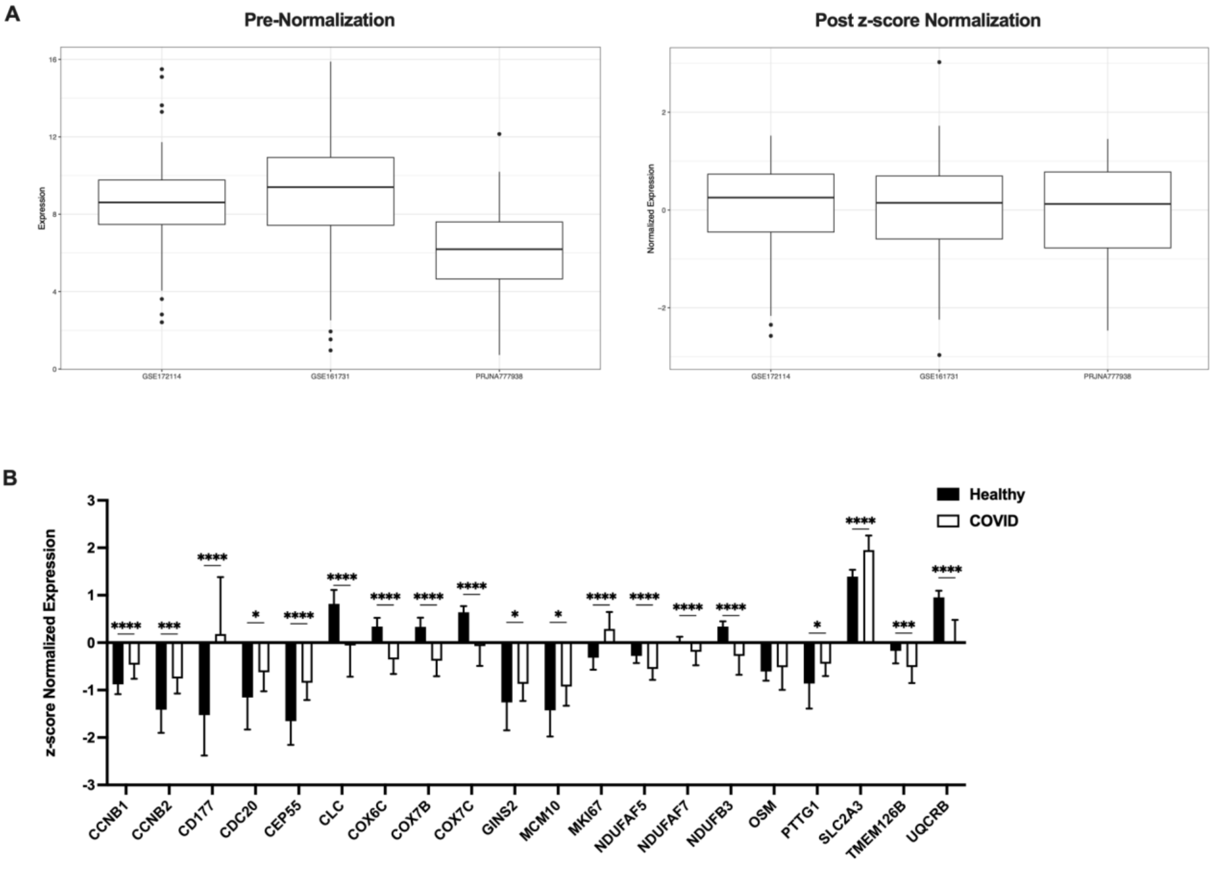

Figure S1: Normalized gene expression values from classification of all COVID patients vs healthy individuals. (**A**) Boxplots of gene expression values for COVID and healthy samples from 3 publicly available datasets pre- and post- z-score normalization. (**B**) Bar graphs comparing normalized expression values of the top 20 gene features resulting from the iterative ML pipeline comparing all COVID patients and healthy individuals. *p<0.05, **p<0.01, ***p<0.001, ****p<0.0001

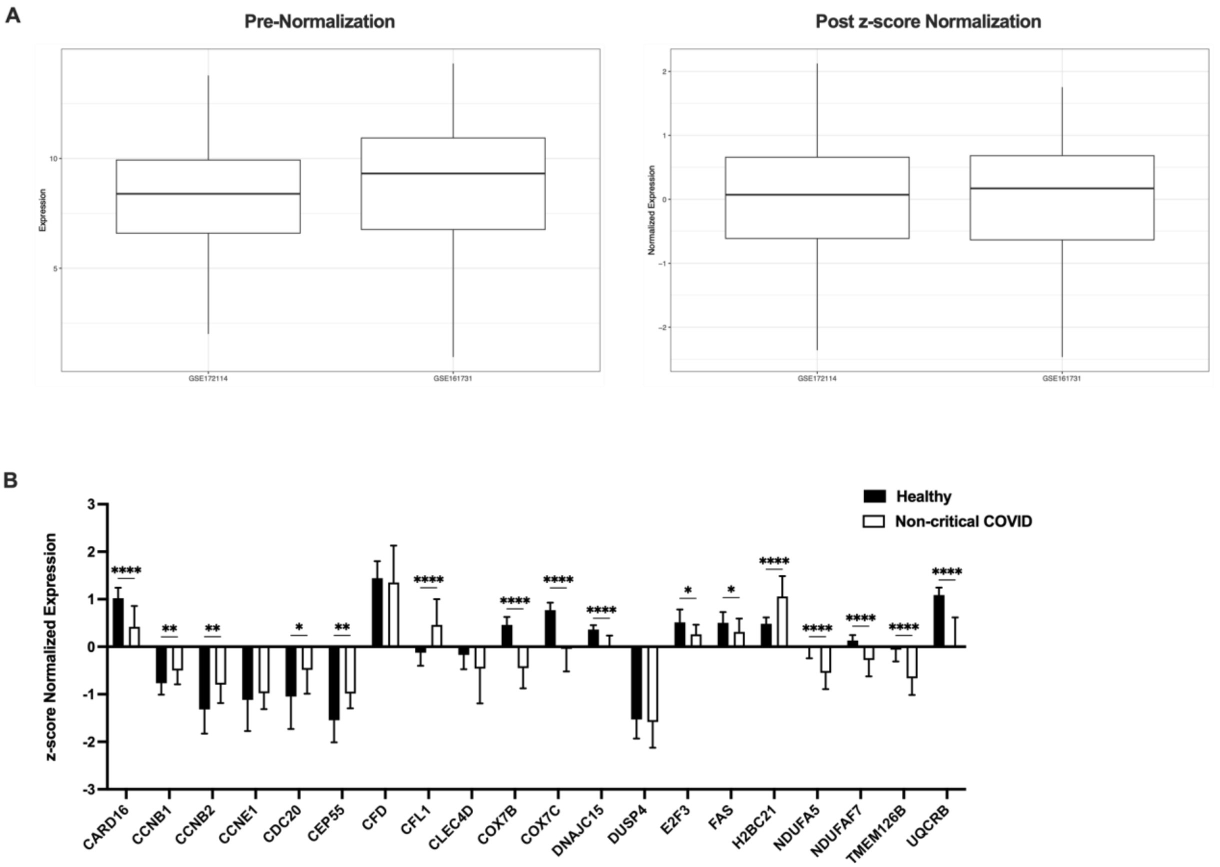

Figure S2: Normalized gene expression values from classification of non-critical COVID patients vs healthy individuals. (**A**) Boxplots of gene expression values for non-critical COVID and healthy samples from 2 publicly available datasets pre- and post- z-score normalization. (**B**) Bar graphs comparing normalized expression values of the top 20 gene features resulting from the iterative ML pipeline comparing non-critical COVID patients and healthy individuals. *p<0.05, **p<0.01, ***p<0.001, ****p<0.0001

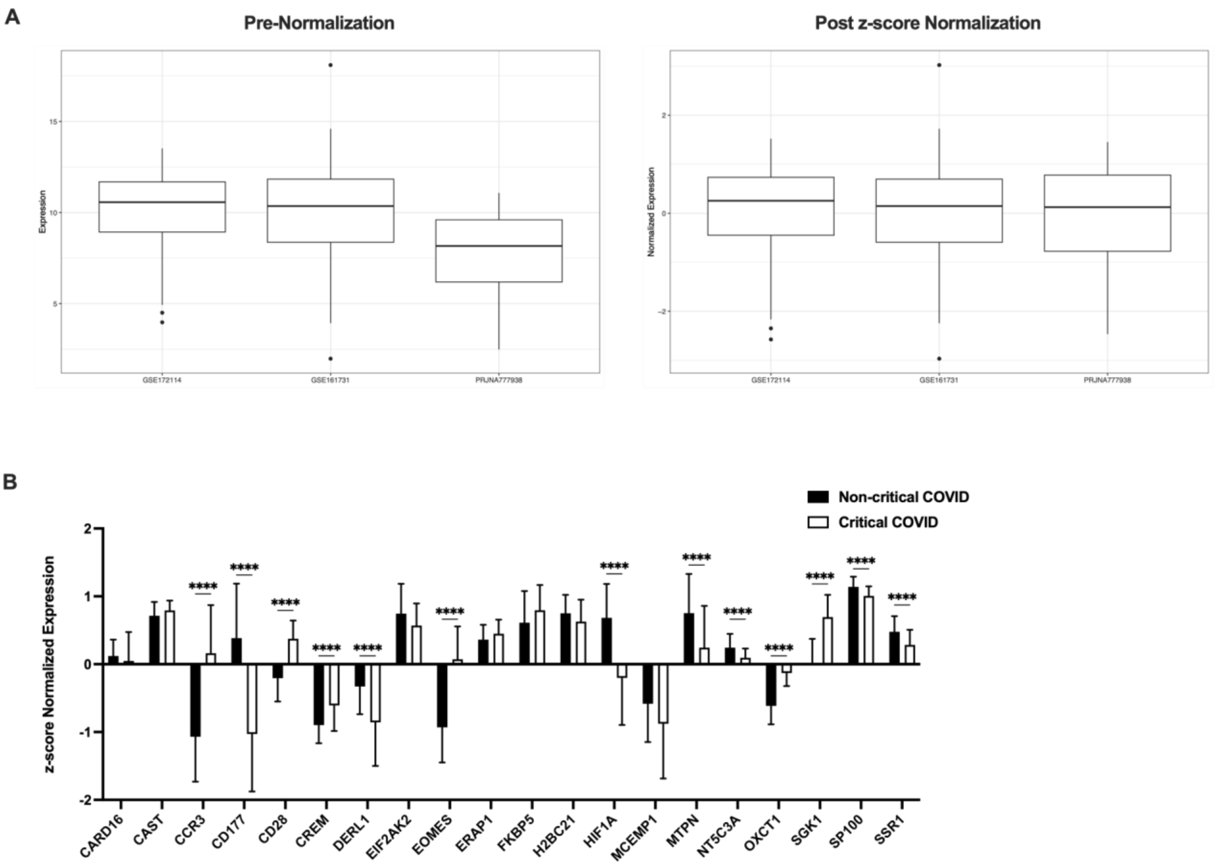

Figure S3: Normalized gene expression values from classification of critical vs non-critical COVID patients. (**A**) Boxplots of gene expression values for critical and non-critical COVID samples from 3 publicly available datasets pre- and post- z-score normalization. (**B**) Bar graphs comparing normalized expression values of the top 20 gene features resulting from the iterative ML pipeline comparing critical and non-critical COVID patients. *p<0.05, **p<0.01, ***p<0.001, ****p<0.0001

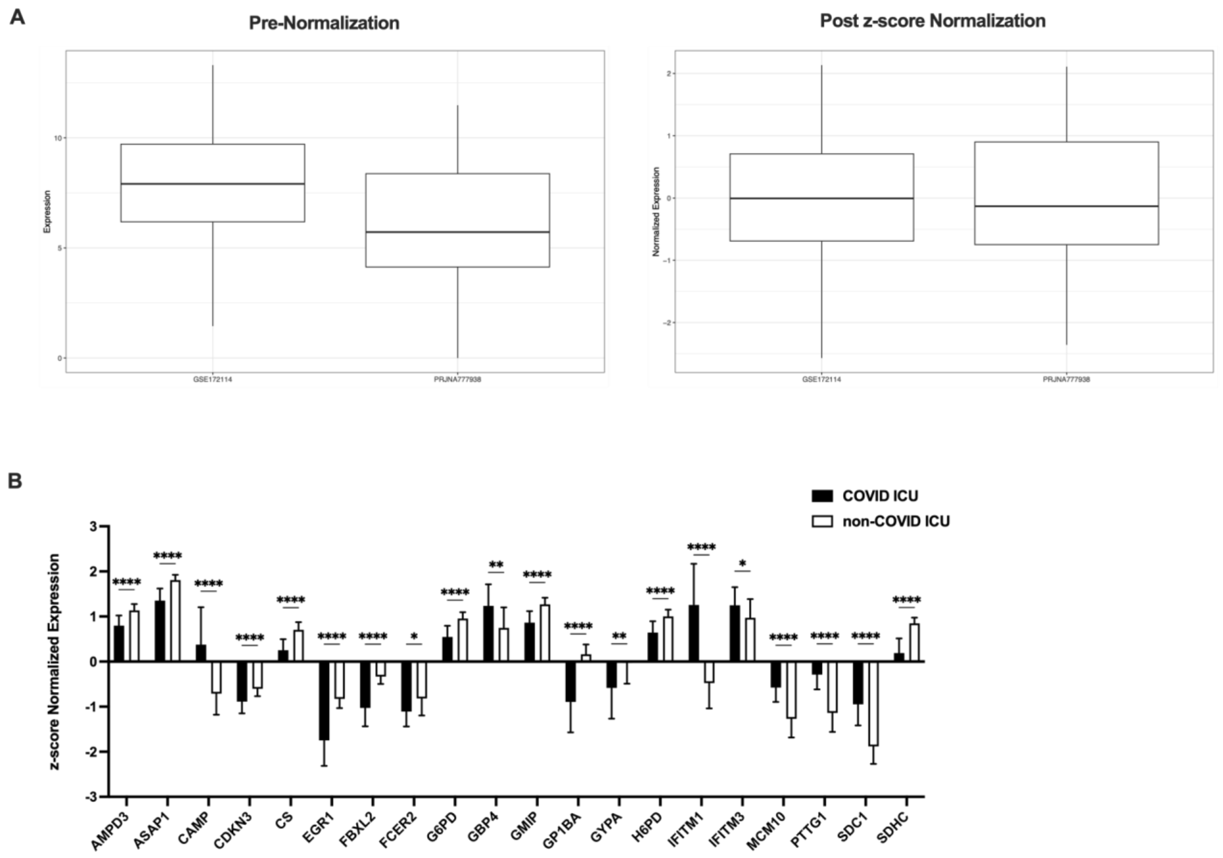

Figure S4: Normalized gene expression values from classification of COVID vs non-COVID ICU patients. (**A**) Boxplots of gene expression values for COVID and non-COVID ICU samples from 2 publicly available datasets pre- and post- z-score normalization. (**B**) Bar graphs comparing normalized expression values of the top 20 gene features resulting from the iterative ML pipeline comparing COVID and non-COVID ICU patients. *$p<0.05$, **$p<0.01$, ***$p<0.001$, ****$p<0.0001$
